# Supplementary material for: Adsorption behaviour and mechanism of the PFOS substitute OBS (sodium p-perfluorous nonenoxybenzene sulfonate) on activated carbon
Source: R Soc Open Sci. 2019 Sep 11;6(9):191069. doi: 10.1098/rsos.191069 (PMC6774934; doi:10.1098/rsos.191069)
Supplement: The properties of ACs and the used kinetics model [file rsos191069supp1.docx]

**Supplementary** **Information for**

**Adsorption behavior and mechanism of the PFOS substitute OBS (****sodium *p*-perfluorous nonenoxybenzene sulfonate) on activated carbon**

Wei Wang^a^, Xin Mi^ab^, Huilan Shi^ab*^, Xue Zhang^ab^, Ziming Zhou^ab^, Chunli Li^a^, Donghai Zhu^a^

^a^ State Key Laboratory of Plateau Ecology and Agriculture, Qinghai University, Xi’ning, Qinghai Province 810016, China

^b^ Eco-environmental engineering college, Qinghai University, Xi’ning, Qinghai Province 810016, China

^*^ Corresponding author, E-mail: [hlshi197701@126.com](mailto:hlshi197701@126.com) (H. Shi)

A manuscript submitted to *R. Soc. open sci.*

**Table S1** Physicochemical properties of OBS

| PFASs | Chemical formula | Molecular lengtha (nm) | Chemical structure |
| --- | --- | --- | --- |
| OBS | C_9_F_17_OC_6_H_4_SO_3_Na | 1.26 |  |

**The mathematical formulas of the pseudo-first-order, pseudo-second-order, Elovich, intra-particle diffusion and Boyed models:**

$\mathrm{Pseudo}-\mathrm{first}-order model:q_{t}=q_{e}\left( 1-e^{\frac{-k_{1}t}{2.303}} \right)$ (1)

$\mathrm{Pseudo}-second-order model: t/q_{t}=1/(k_{2}{q_{e}^{2})+t/q}_{e}=1/v_{0}+{\frac{t}{q}}_{e}$ (2)

$Elovich model:q_{t}=\frac{1}{b}\ln\left( abt \right)$ (3)

$Intra-particle diffusion model:q_{t}=k_{ni}t^{\frac{1}{2}}+c_{ni}$ (4)

Boyd model: $f=\frac{q_{e}}{q_{t}} f>0.85 Bt=-0.4977-\ln\left( 1-f \right) ; f<0.85 Bt={(\sqrt{\pi}-\sqrt{\pi-\left( \frac{\pi^{2}f}{3} \right)})}^{2}$ (5)

where q_t_ (mmol/g) and q_e_ (mmol/g) are the amount adsorbed at time t and at equilibrium, respectively; k_1_ (h^-1^), k_2_ (mmol^-1^ g h^-1^) and k_ni_ (mmol g^-1^ h^-0.5^) are the rate constants of the pseudo-first-order, pseudo-second-order and intra-particle diffusion models, respectively; a and b are the Elovich model adsorption rate constants; c_ni_ (mmol g^-1^) is the boundary layer thickness coefficient. Eqs. (5) can be used in predicting the mechanism of the adsorption process. This is done by plotting Bt against time, if the plot is linear and passes through the origin then pore-diffusion controls the rate of mass transfer. If the plot is nonlinear or linear but does not pass through the origin, then it is concluded that film-diffusion or chemical reaction control the adsorption rate.

**Table S2** Calculated constants of the kinetic models for OBS adsorption on seven ACs

| Models | parameters | GAC | PAC | UAC | R1-GAC | R2-GAC | O1-GAC | O2-GAC |
| --- | --- | --- | --- | --- | --- | --- | --- | --- |
|  | q_e_ | 0.052 | 0.205 | 0.134 | 0.068 | 0.183 | 0.0373 | 0.041 |
| pseudo-first-order | k_1_ | 4.260 | 0.504 | 3.491 | 2.546 | 0.187 | 2.320 | 0.585 |
|  | R^2^ | 0.168 | 0.888 | 0.771 | 0.559 | 0.760 | 0.856 | 0.727 |
|  | q_e_ | 0.055 | 0.221 | 0.141 | 0.074 | 0.206 | 0.040 | 0.044 |
| pseudo-second-order | v_0_ | 0.1261 | 0.0742 | 0.3283 | 0.0943 | 0.0235 | 0.056 | 0.0191 |
|  | R^2^ | 0.417 | 0.943 | 0.948 | 0.783 | 0.825 | 0.945 | 0.847 |
|  | a | 2.222 | 0.289 | 30.99 | 0.813 | 0.133 | 0.897 | 0.085 |
| Elovich model | b | 164.1 | 28.21 | 79.84 | 107.6 | 32.48 | 223.4 | 148.6 |
|  | R^2^ | 0.838 | 0.956 | 0.910 | 0.973 | 0.876 | 0.839 | 0.924 |
|  | k_n1_ | 0.0404 | 0.0487 | 0.0481 | 0.0297 | 0.0303 | 0.014 | 0.008 |
| Intra-particle diffusion | C_n1_ | 0.0017 | 0.0133 | 0.0277 | 0.0072 | 0.0133 | 0.005 | 0.006 |
|  | R^2^ | 0.883 | 0.974 | 0.748 | 0.780 | 0.810 | 0.841 | 0.932 |

**Fig.S1** the distribution of particle size of UAC

**Fig. S2** Pore size distribution of different activated carbons (dV(D): the derivative of pore volume (V) with respect to pore size (D))

**Fig. S3** FTIR spectra of R1-GAC and R2-GAC

**Fig. S4** Adsorption kinetics of OBS on different activated carbons as well as modeling using the intra-particle diffusion kinetic model

**Fig. S5** Adsorption kinetics of OBS on different activated carbons as well as modeling using the Boyd model

**Fig. S6** D-R isotherm plots for the adsorption of OBS onto GAC (a), PAC (b), UAC (c), R1-GAC, R2-GAC, O1-GAC and O2-GAC (d)
