# Supplementary material for: Adsorption behaviour and mechanism of the PFOS substitute OBS (sodium p-perfluorous nonenoxybenzene sulfonate) on activated carbon
Source: R Soc Open Sci. 2019 Sep 11;6(9):191069. doi: 10.1098/rsos.191069 (PMC6774934; doi:10.1098/rsos.191069)
Supplement: Original experiment data [file rsos191069supp2.docx]

**All the experiment data can be accessed by Origin software.**

**Fig. 2.**

**

**Fig. 3.**

**

**Fig. 4.**

**

**Fig. 5.**

**Fig. 6.**

**Fig.S1.**

**Fig. S2.**

**Fig. S3.**

**Fig.S4.**

**Fig. S5**
